# Workplace Violence in Nursing: Prevalence and Nature of Incidents in a Tertiary University Hospital

**DOI:** 10.3390/ijerph23070932

**Published:** 2026-07-21

**Authors:** Fábio Silva Rosa, Mara Ambrosina de Oliveira Vargas, Daiane Dal Pai, Rosinei Nascimento Ferreira, Davydson Gouveia Santos, Rafael Limeira de Freitas, Maria de Nazaré de Souza Ribeiro, Laura Cavalcanti de Farias Brehmer, José Luís Guedes dos Santos, Elaine Cristina Novatzki Forte

**Affiliations:** 1 Programa de Pós-Gradução em Enfermagem, Universidade Federal de Santa Catarina, Florianópolis 88040-900, SC, Brazil; fabiorosa18@gmail.com (F.S.R.); ambrosina.mara@ufsc.br (M.A.d.O.V.); davydson_gs@hotmail.com (D.G.S.); rafaellimeira@outlook.com (R.L.d.F.); laura.brehmer@ufsc.br (L.C.d.F.B.); jose.santos@ufsc.br (J.L.G.d.S.); elaine.forte@ufsc.br (E.C.N.F.); 2 Programa de Pós-Gradução em Enfermagem, Universidade Federal do Rio Grande do Sul, Porto Alegre 90620-110, RS, Brazil; daiane.dalpai@gmail.com; 3 Programa de Pós-Graduação em Enfermagem em Saúde Pública, Universidade do Estado do Amazonas, Manaus 69065-001, AM, Brazil; mnribeiro2@gmail.com

**Keywords:** working conditions, nursing, occupational stress, occupational risks, occupational health, workplace violence

## Abstract

**Highlights:**

**Public health relevance—How does this work relate to a public health issue?**

- Workplace violence in nursing is a major public health issue affecting workers’ health and patient care quality.
- Psychological and verbal violence contribute to unsafe and unhealthy work environments.

**Public health significance—Why is this work of significance to public health?**

- The study shows that more than 70% of nursing professionals reported experiencing workplace violence, highlighting the magnitude of the phenomenon and the urgent need for institutional actions and public policies aimed at protecting healthcare workers.
- The findings demonstrate significant consequences, including work leave, medication use, psychological treatment, and intentions to resign from the profession, indicating substantial repercussions for the healthcare workforce and continuity of care.

**Public health implications—What are the key implications or messages for practitioners, policy makers and/or researchers in public health?**

- Hospitals should strengthen violence-prevention policies, reporting systems, and psychological support.
- Public health strategies are needed to promote safe workplaces and protect the nursing workforce.

**Abstract:**

The cumulative occurrence of workplace violence in nursing represents a growing challenge to workers’ health and the quality of care. This study aimed to analyze the prevalence, characteristics, and repercussions of workplace violence among nursing professionals in a tertiary university hospital. A cross-sectional, exploratory, and descriptive study was conducted with 573 nursing professionals using an electronic questionnaire for data collection. Descriptive and inferential analyses were performed using Pearson’s chi-square test with Bonferroni correction. Overall, 71.2% of participants reported ever experiencing workplace violence while working at the hospital, according to the questionnaire. Among those exposed, workplace bullying/intimidation (76.5%), verbal violence (49.8%), and psychological violence (43.9%) were the most frequently reported forms of violence. Physical violence (8.1%), sexual violence (5.6%), racial discrimination (9.1%), gender discrimination (6.4%), ageism (7.8%), and ableism (2.0%) were reported less frequently. Managers/supervisors (37.3%), patients’ family members or companions (29.4%), and coworkers (28.4%) were identified as the main perpetrators. Participants who experienced workplace violence reported adverse occupational and health-related outcomes, including medication use, psychological treatment, work absences, intention to resign (50.2%), and intention to leave the profession (42.6%). Underreporting was substantial, with only 33.7% of incidents being formally reported. Workplace violence was highly prevalent among nursing workers and was accompanied by self-reported adverse consequences for health, well-being, and professional performance. These findings underscore the need for effective institutional strategies aimed at preventing, monitoring, and addressing workplace violence in hospital settings.

## 1. Introduction

Violence has emerged as an expanding phenomenon, often trivialized in the daily routine of healthcare services in Brazil. It remains a problem that is still insufficiently quantified, classified, or properly understood, and is sometimes perceived by professionals as an inherent risk of their work practice [1]. The World Health Organization (WHO) defines violence as the intentional use, or threat of use, of physical force against another person or against oneself, which results or is likely to result in injury, death, or psychological harm [2].

In the health sector, the document Framework Guidelines for Addressing Workplace Violence in the Health Sector was developed through a collaboration between the International Labor Organization (ILO), the International Council of Nurses (ICN), the World Health Organization (WHO), and Public Services International (PSI). It provides global guidelines for understanding and addressing workplace violence in healthcare services. In this document, workplace violence is defined as incidents in which workers are abused, threatened, or assaulted in circumstances related to their professional activities, including commuting to and from work, constituting an explicit or implicit challenge to their safety, health, and well-being [3].

The ILO, in turn, in its Convention on Violence and Harassment (2019), defines violence and harassment in the world of work as a range of unacceptable behaviors and practices, or threats thereof, occurring once or repeatedly, that aim at, result in, or are likely to result in physical, psychological, sexual, or economic harm [4].

According to the WHO, between 8% and 38% of healthcare professionals experience physical violence at some point in their careers, while an even larger number are exposed to verbal aggression or threats [5]. In the case of nursing, verbal and physical violence, as well as threats, occur so frequently that, in many contexts, they are already perceived as an inherent part of the job. This process of normalization contributes to the underreporting of violent incidents [6].

Despite international recognition of violence as a serious occupational problem, its magnitude remains underestimated in Brazil, marked by high levels of underreporting and by the normalization of such episodes in the daily routine of healthcare services. National studies indicate that the phenomenon is still insufficiently explored in terms of measurement and classification [7].

The situation becomes even more concerning among nursing professionals. A survey conducted by the Regional Nursing Council of São Paulo (“COREN-SP”) in 2023 revealed that 80% of professionals had already experienced some type of aggression in the workplace, with 90% of these incidents being verbal in nature. Additionally, 48% reported having been assaulted more than once [8]. In the Federal District, a study conducted by “COREN-DF” in 2022 found that 82.7% of professionals had experienced physical violence, with at least one verbal or physical aggression occurring daily [9].

These data highlight the magnitude of the phenomenon and the difficulty in adopting effective prevention and response strategies. The nursing team, due to its frontline role in care, direct and continuous contact with patients and families, and long working hours, stands out as one of the most vulnerable groups. A study conducted in a public hospital in southern Brazil revealed that 15.2% of healthcare professionals had experienced physical violence, while 48.7% had been victims of psychological violence, including verbal aggression (35.4%), moral harassment (24.9%), racial discrimination (8.7%), and sexual harassment (2.5%) [10].

Another study, carried out in hospital units of the 8th Health Region of Paraná, found that 44% of nursing professionals had experienced some type of workplace violence in the previous 12 months. Verbal violence was the most prevalent (47.7%), followed by physical violence (11.9%) and sexual harassment (2.8%) [11].

Moral harassment and verbal violence in the hospital environment are therefore frequent realities for nursing professionals, manifesting mainly through humiliation and verbal aggression perpetrated by hierarchical superiors or coworkers. Their consequences include psychological harm such as anxiety, depression, feelings of incapacity, and loss of interest in the profession [12].

The repercussions of workplace violence go beyond immediate physical harm, affecting workers’ productivity, mental and physical health, and social relationships. In addition, such violence compromises professional safety and the quality of care provided, weakening processes aimed at humanizing healthcare. Given this scenario, the urgency of implementing institutional strategies for prevention, psychological support, and disciplinary measures to minimize the impacts of violence in the hospital context becomes evident [12].

Thus, it is essential to deepen the understanding of the prevalence, manifestations, and consequences of workplace violence in the hospital environment, especially among nursing professionals. In this regard, the present study aims to analyze the prevalence, characteristics, and repercussions of workplace violence among nursing professionals working in a tertiary-level university hospital in southern Brazil.

## 2. Materials and Methods

### 2.1. Study Design

To analyze the prevalence, characteristics, and repercussions of workplace violence among nursing professionals in the hospital setting, a quantitative, cross-sectional, exploratory, and descriptive study was conducted in a large university hospital located in southern Brazil. The institution has 890 beds and provides healthcare services predominantly within the Brazilian Unified Health System (Sistema Único de Saúde—SUS).

### 2.2. Participant Recruitment

Between 12 August and 9 September 2024, a sample of nursing professionals employed at the institution was recruited. Eligible participants included registered nurses, nursing technicians, and nursing assistants with an active employment contract and at least one year of professional experience at the hospital. This criterion was adopted to ensure familiarity with the organizational environment, work processes, and institutional practices.

The eligible population consisted of 2518 professionals distributed across different healthcare units. All eligible professionals were invited to participate through institutional email, with support from the Nursing Directorate.

Sample size was calculated using OpenEpi software, version 3.01, considering a finite population of 2518 professionals, a 95% confidence level, a 4% margin of error, and an expected prevalence of 50%, resulting in a minimum required sample of 503 participants. At the end of the data collection period, 573 professionals had completed the questionnaire, corresponding to a response rate of 22.8%.

Data were collected through an electronic questionnaire. The informed consent form was made available on the digital platform, and access to the questionnaire was granted only after participants provided electronic consent. Participants were informed about the study objectives, estimated completion time (15–20 min), voluntary participation, and their right to withdraw from the study at any time without any consequences.

### 2.3. Data Collection Procedures

Data were collected using a structured questionnaire specifically developed for this study based on national and international literature on workplace violence among healthcare professionals, including studies addressing different types of violence, associated factors, and consequences for workers. The questionnaire was also informed by reference documents from the World Health Organization (WHO), the International Labour Organization (ILO), and the International Council of Nurses (ICN), which provide guidance on the surveillance and prevention of workplace violence in healthcare settings [1,2,3,5,10].

The instrument was administered through an electronic form developed using Google Forms^®^ and organized into four sections: (1) participants’ sociodemographic characteristics, including sex, age, race/ethnicity, marital status, sexual orientation, and disability status. Race/ethnicity was self-reported and categorized according to the classification adopted by the Brazilian Institute of Geography and Statistics (IBGE); (2) occupational characteristics, including professional category, work unit, work shift, educational level, and professional experience; (3) workplace violence experiences, including occurrence, frequency, and types of violence (physical, psychological, verbal, workplace bullying/intimidation, sexual violence, and discrimination), characteristics of perpetrators, and perceptions of hostility in the work environment; and (4) repercussions of workplace violence, including health consequences, use of support services, reporting behaviors, fear of retaliation, and intention to leave the institution or the profession.

Workplace violence was assessed using questions beginning with the expression “Have you ever experienced…”, referring to participants’ experiences while working at the institution. Therefore, the reported prevalence reflects cumulative exposure during employment at the institution rather than events occurring within a specific recall period (e.g., the previous 12 months).

To standardize participants’ understanding and minimize potential overlap among categories, brief operational definitions of each type of violence were provided before the corresponding questions. These definitions were developed based on WHO and ILO documents, as well as studies addressing workplace violence in nursing and healthcare settings [1,2,3,5,10]. Physical violence was defined as the use of physical force against a worker; verbal violence as insults, offensive language, threats, shouting, or aggressive communication; psychological violence as actions causing emotional distress, intimidation, embarrassment, or humiliation; workplace bullying/intimidation as repeated exposure to hostile, humiliating, offensive, or degrading behaviors in the workplace; sexual violence as unwanted sexual comments, advances, or behaviors; and discrimination as unfair or unfavorable treatment related to race/ethnicity, gender, age, or disability. These definitions were made available to participants before the workplace violence questions to guide the classification of experienced events.

The questionnaire’s content validity was qualitatively assessed by a panel of five experts with experience in occupational health, workplace violence, hospital nursing, and research methodology. Subsequently, a pilot study was conducted with 10 nursing professionals presenting characteristics similar to those of the target population. The pilot study evaluated the clarity, relevance, and comprehensibility of the questions, as well as the semantic adequacy of the instrument and the functionality of the electronic data collection platform. Suggestions arising from this process were incorporated into the final version of the questionnaire, and no limitations compromising its applicability were identified.

Because the instrument was designed to identify events, characteristics, and consequences of workplace violence rather than to measure a latent psychometric construct, internal consistency analyses were not performed. No duplicate questionnaires were identified, and all analyzed records were complete.

### 2.4. Data Analysis

Data were analyzed using the Statistical Package for the Social Sciences (SPSS), version 26.0 (IBM Corp., Armonk, NY, USA). Descriptive statistics were calculated, including absolute and relative frequencies for categorical variables and means and standard deviations for continuous variables.

The primary dependent variable was self-reported ever experiencing workplace violence during employment at the institution (yes/no). Characteristics, types, and repercussions of workplace violence among exposed participants were also analyzed. The investigated forms of violence included physical, psychological, verbal, workplace bullying/intimidation, sexual violence, and discrimination. Sociodemographic and occupational variables included sex, age, race/ethnicity, marital status, sexual orientation, disability status, professional category, work unit, work shift, educational level, and professional experience. Repercussions of violence were assessed through variables related to incident reporting, fear of retaliation, use of occupational health services, work leave, medication use, psychological treatment, and intention to leave the institution or profession.

The proportion of participants reporting ever experiencing workplace violence was calculated considering the total sample (*n* = 573). The proportions of participants reporting each specific type of violence, perpetrator profiles, and violence-related repercussions were calculated among participants who reported ever experiencing workplace violence (*n* = 408), unless otherwise specified in the tables.

Associations between workplace violence variables and study outcomes were initially tested using Pearson’s chi-square test (χ^2^). When variables contained more than two categories and the overall chi-square test was statistically significant, post hoc pairwise comparisons with Bonferroni correction were performed. Statistical significance was established at *p* < 0.05. To improve the readability of the results, only statistically significant associations are presented; however, complete analyses were performed for all investigated variables.

### 2.5. Ethical Considerations

This study is part of the project “Workplace Violence in Hospital Nursing: A Mixed-Methods Study,” funded by the Brazilian Coordination for the Improvement of Higher Education Personnel (CAPES). The study was approved by the Research Ethics Committee of the Federal University of Santa Catarina (UFSC) (Approval No. 7241146; CAAE No. 80554324.7.3001.0121). To ensure participant protection, all measures related to anonymity and confidentiality were implemented. Electronic informed consent was obtained from all participants prior to questionnaire completion, in accordance with the principles of the Declaration of Helsinki.

## 3. Results

A total of 573 nursing professionals participated in the study, the majority of whom were women (81.3%), with a mean age of 43.95 years (SD = 8.44). Nursing technicians represented 61.8% of the sample, and the average length of professional experience was 19.32 years. Regarding academic qualifications, specialization was the most frequently reported highest academic degree, followed by a master’s degree and doctorate. Complete sample characteristics are presented in Table 1.

### 3.1. Prevalence and Types of Workplace Violence

Overall, 71.2% of participants reported ever experiencing workplace violence during their employment at the institution. Workplace bullying/intimidation (76.5%), verbal violence (49.8%), and psychological violence (43.9%) were the most frequently reported forms of violence, whereas physical violence (8.1%), sexual harassment (5.6%), and discrimination were less common. Table 2 summarizes the distribution of the different types of workplace violence.

### 3.2. Consequences of Violence

Among the 408 professionals who reported workplace violence, fear of retaliation was the most frequent consequence (70.7%), followed by consideration of resigning from the institution (50.0%), psychological treatment (47.3%), consideration of changing profession (42.4%), and medication use (31.9%). Formal reporting of incidents was observed in only 33.7% of cases, while only 25.4% of participants reported feeling protected when reporting workplace violence. Moral harassment and psychological violence were the types most consistently associated with adverse occupational and health-related outcomes (Table 3).

### 3.3. Profile of the Aggressors

The main external aggressors were family members/companions (29.4%) and patients (18.4%). Among internal aggressors, leadership (37.3%), coworkers (28.4%), and immediate supervisors (26.2%) stood out. When the aggressor was a member of the healthcare team, nurses (44.9%) were the most frequently reported (Table 4).

### 3.4. Hostility Perception

Hostility in the work environment was perceived by 65.6% of professionals and showed a significant association with experiencing violence (79.9% vs. 54.3%; *p* < 0.001) and witnessing violence (83.5% vs. 64.5%; *p* < 0.001), as shown in Table 5.

## 4. Discussion

This study showed that more than 70% of nursing professionals reported ever experiencing workplace violence during their employment at the institution, with workplace bullying/intimidation, verbal violence, and psychological violence being the most frequently reported forms. These findings are consistent with national and international investigations that identify nursing as one of the professional categories most exposed to occupational violence, whether due to frontline positioning, close contact with patients and families, or the hierarchical structure of healthcare services [10,11,12,13]. In various contexts, non-physical violence tends to surpass physical violence, influenced by organizational climate, work overload, and managerial styles [10,11,14].

In the present study, moral harassment emerged as the most frequent type of violence and the one with the greatest number of significant associations with negative outcomes, such as illness, medication use, work leave, and intention to resign. This pattern is consistent with studies reporting associations between moral harassment, occupational stress, burnout, adverse care indicators, professional identity, patient safety, and organizational climate [12,15,16].

Verbal and psychological violence also showed a strong association with emotional distress and turnover intention, findings consistent with studies indicating that such forms of violence have been associated with moderate to severe stress, lower job satisfaction, increased care errors, and greater likelihood of work leave [17,18]. In particular, psychological violence has been identified as a risk factor for increased turnover intention, mediated by reduced professional satisfaction [18].

Although less frequent, physical violence, sexual harassment, and discrimination reveal important vulnerabilities. Research indicates that physical episodes may involve significant risk, including assaults with objects and the need for medical care and work leave [19,20]. In the case of sexual violence, recent studies show a growing trend, often perpetrated by patients, reinforcing the persistent risk scenario for nurses [21]. Racial, gender, age-based, and ableist discrimination emerge as forms of institutionalized violence, often rendered invisible and frequently associated with lower well-being, reduced engagement, and less favorable perceptions of organizational support [22,23,24,25]. This set of practices reinforces structural inequalities and undermines the retention of workers belonging to minority groups.

Among the reported repercussions, the use of medication, seeking psychological treatment, work leave, and intention to resign stood out, suggesting that workplace violence was associated with a substantial self-reported physical and psychological burden among participants. The literature also reports associations between repeated exposure to workplace violence, higher levels of burnout, and a greater likelihood of care-related errors, which may negatively affect the quality and safety of care [26].

Underreporting, identified in this study, reflects a global trend. Research shows that most episodes remain unreported due to fear of retaliation, institutional distrust, or the normalization of violence in the hospital environment [14,27,28]. These factors may contribute to underestimating the magnitude of the phenomenon and may hinder the development of effective prevention strategies. The perception of a hostile environment, which was significantly associated with experiencing and witnessing violence, may reflect the importance of organizational climate as a contextual factor related to workplace violence, as previously reported in Brazilian and international studies [10,11,29].

From an organizational perspective, the findings reinforce the need for robust and multifaceted institutional policies. Strategies such as accessible reporting systems, leadership training, clear response protocols, psychological support for victims, and accountability mechanisms are recommended by international organizations, including the ILO and WHO [3,4]. Effective interventions should involve continuous actions, monitoring of indicators, and active participation of teams, promoting psychologically safe environments aligned with occupational health prevention principles.

This study has several limitations that should be considered. Data were collected from a single university hospital, which may limit the generalizability of the findings to other healthcare settings. In addition, the use of self-reported information may be subject to recall bias and social desirability bias. Voluntary participation through an electronic questionnaire may also have introduced selection bias, as professionals who were more affected by or sensitized to the issue may have been more likely to participate in the study. Furthermore, the cross-sectional design does not allow causal relationships to be established between exposure to workplace violence and its reported repercussions. Therefore, the reported findings should be interpreted as associations rather than evidence of causal relationships. Finally, multivariate analyses were not performed because the primary objective of the study was to describe the prevalence, characteristics, and repercussions of workplace violence rather than to identify independent predictors associated with its occurrence.

## 5. Conclusions

More than 70% of nursing professionals reported ever experiencing workplace violence during their employment at the institution, which was associated with self-reported negative repercussions for health, well-being, professional performance, and job retention. Workplace bullying/intimidation, verbal violence, and psychological violence were the most frequently reported forms, whereas discrimination and physical and sexual violence, although less frequent, revealed persistent vulnerabilities.

The reported repercussions, including illness, medication use, seeking psychological care, and the intention to resign or change professions, were associated with workplace violence and highlight its potential implications for the nursing workforce. Underreporting and the perception of a hostile environment were frequently reported and may reflect institutional barriers that hinder effective prevention and response strategies.

The findings support the importance of strengthening institutional policies, reporting systems, and prevention and support strategies aimed at promoting safer work environments for nursing professionals. Although causal inferences cannot be drawn from this cross-sectional study, the observed associations reinforce the relevance of organizational initiatives to prevent workplace violence and support affected workers.

## Figures and Tables

**Table 1:** Sample Characterization (*n* = 573). Florianópolis, SC, Brazil, 2024.

Sample Characterization	*n* (573)	%
Gender		
Female	466	81.3%
Male	107	18.7%
Age		
20–30 years	31	5.4%
31–40 years	162	28.3%
41–50 years	261	45.5%
Over 50 years	119	20.8%
Sexual orientation		
Heterosexual	540	94.2%
Lesbian	7	1.2%
Gay	16	2.8%
Bisexual	9	1.6%
Asexual	1	0.2%
Marital Status		
Single	140	24.4%
Married/lives with partners	351	61.3%
Divorced/Widow	70	12.2%
Other	12	2.1%
Skin Color		
White	450	78.5%
Yellow	2	0.3%
Brown	58	10.1%
Black	63	11%
Disability?		
Yes	29	5.1%
No	544	94.9%
Category		
Nurse	198	34.6%
Nursing Technician	354	61.8%
Nursing Assistant	21	3.7%
Work shift		
Morning	133	23.2%
Afternoon	183	31.9%
Night	205	35.8%
Intermediate	14	2.4%
Day-off substitute	38	6.7%
Highest academic degree		
Non-nurses	362	63.2%
Graduation	9	1.6%
Specialization	111	19.4%
Master’s degree	71	12.4%
Doctorate	18	3.1%
Postdoctoral training	2	0.3%

**Table 2:** Distribution of workplace violence and specific types of violence among nursing professionals. Florianópolis, SC, Brazil, 2024.

Types and Frequencies of Violence	*n*	%
Have you ever experienced violence in the workplace?		
Yes	408	71.2%
No	165	28.8%
Have you ever experienced moral harassment/intimidation?		
Yes	312	76.5%
No	96	23.5%
Have you ever experienced verbal violence?		
Yes	203	49.8%
No	205	50.2%
Have you ever experienced psychological violence?		
Yes	179	43.9%
No	229	56.1%
Have you ever experienced physical violence?		
Yes	33	8.1%
No	375	91.9%
Have you ever experienced sexual harassment/violence?		
Yes	23	5.6%
No	385	94.4%
Have you ever experienced racism?		
Yes	37	9.1%
No	371	90.9%
Have you ever experienced gender discrimination?		
Yes	26	6.4%
No	382	93.6%
Have you ever experienced age discrimination?		
Yes	32	7.8%
No	376	92.2%
Have you ever experienced ableism?		
Yes	8	2.0%
No	400	98.0%

Note: Overall prevalence refers to participants who reported ever experiencing workplace violence during their employment at the institution (*n* = 573). The proportions of specific types of violence were calculated among participants who reported ever experiencing workplace violence (*n* = 408).

**Table 3:** Consequences of workplace violence and significant association with the type of violence (*n* = 408). Florianópolis, SC, Brazil, 2024.

Consequences	Yes *n*	No *n*	Significant Associations (χ^2^; df; *p*)
	%	%	
Felt protected When reporting	104 (25.4)	304 (74.6)	Moral harassment (χ^2^ = 9.09; df = 1; *p* = 0.003); Verbal violence (χ^2^ = 4.69; df = 1; *p* = 0.030); Sexual violence/harassment (χ^2^ = 4.22; df = 1; *p* = 0.040)
Fear of retaliation	287 (70.7)	119 (29.3)	Moral harassment (χ^2^ = 22.73; df = 1; *p* < 0.001); Psychological violence (χ^2^ = 8.74; df = 1; *p* = 0.003); Racial discrimination (χ^2^ = 4.90; df = 1; *p* = 0.027); Gender discrimination (χ^2^ = 6.26; df = 1; *p* = 0.012)
Reported the incident	137 (33.7)	271 (66.3)	No statistically significant associations
Sought Occupational Health Services	97 (23.8)	311 (76.2)	Moral harassment (χ^2^ = 11.81; df = 1; *p* < 0.001); Psychological violence (χ^2^ = 4.68; df = 1; *p* = 0.030); Physical violence (χ^2^ = 4.74; df = 1; *p* = 0.029)
Work leave (medical certificate)	101 (24.8)	307 (75.2)	Moral harassment (χ^2^ = 15.32; df = 1; *p* < 0.001); Psychological violence (χ^2^ = 14.50; df = 1; *p* < 0.001); Physical violence (χ^2^ = 8.13; df = 1; *p* = 0.004)
Work leave (social security)	34 (8.3)	374 (91.7)	Moral harassment (χ^2^ = 11.18; df = 1; *p* < 0.001); Physical violence (χ^2^ = 7.71; df = 1; *p* = 0.005); Sexual violence/harassment (χ^2^ = 15.46; df = 1; *p* < 0.001)
Medication treatment	130 (31.9)	278 (68.1)	Moral harassment (χ^2^ = 23.19; df = 1; *p* < 0.001); Psychological violence (χ^2^ = 17.78; df = 1; *p* < 0.001)
Psychological treatment	193 (47.3)	215 (52.7)	Moral harassment (χ^2^ = 31.13; df = 1; *p* < 0.001); Psychological violence (χ^2^ = 35.84; df = 1; *p* < 0.001)
Other treatments	50 (12.3)	358 (87.7)	Moral harassment (χ^2^ = 7.35; df = 1; *p* = 0.007)
Considered resigning	204 (50.0)	204 (50.0)	Moral harassment (χ^2^ = 38.10; df = 1; *p* < 0.001); Psychological violence (χ^2^ = 17.72; df = 1; *p* < 0.001)
Considered changing professions	173 (42.4)	235 (57.6)	Moral harassment (χ^2^ = 25.09; df = 1; *p* < 0.001); Psychological violence (χ^2^ = 38.57; df = 1; *p* < 0.001); Racial discrimination (χ^2^ = 6.36; df = 1; *p* = 0.012)

Note: Percentages were calculated considering only participants who reported workplace violence (*n* = 408). Pearson’s chi-square test (χ^2^) was used to evaluate associations between types of workplace violence and reported consequences. Values presented in the last column correspond to chi-square statistics (χ^2^), degrees of freedom (df), and *p*-values. Reported *p*-values are unadjusted Pearson’s chi-square test results. All investigated associations were tested using Pearson’s chi-square test. For presentation purposes, only statistically significant associations (*p* < 0.05) are shown. Complete analyses were performed for all investigated variables. Bonferroni correction was applied exclusively to post hoc analyses involving variables with more than two categories.

**Table 4:** Types of aggressors reported by participants who were victims of workplace violence (*n* = 408). Florianópolis, SC, Brazil, 2024.

Category of the Aggressor	Yes (*n*)	Yes (%)	No (*n*)	No (%)
External aggressors				
Family members/companions	120	29.4%	288	70.6%
Patients	75	18.4%	333	81.6%
Internal aggressors—hierarchical				
Unit leadership/management	152	37.3%	256	62.7%
Immediate supervisor	107	26.2%	301	73.8%
Internal aggressors—work colleagues				
Work colleagues	116	28.4%	292	71.6%
Internal aggressors—other health professionals				
Nurses	183	44.9%	225	55.1%
Nursing technicians	88	21.6%	320	78.4%
Physicians	86	21.1%	322	78.9%
Psychologists	3	0.7%	405	99.3%
Physical therapists	2	0.5%	406	99.5%
Nutritionists	2	0.5%	406	99.5%
Speech therapists	0	0.0%	408	100%
Other	5	1.2%	403	98.8%
Internal aggressors—scholars				
Professors	29	7.1%	379	92.9%

**Table 5:** Perception of hostility in the workplace and its association with workplace violence among nursing professionals (*n* = 573). Florianópolis, SC, Brazil, 2024.

**Hostility Perception**	**Experienced Violence—*n* (%)**	**Have not experienced violence—*n* (%)**	**χ^2^ (df)**	***p***
Yes, *n* = 376 (65.6%)	301 (79.9%)	75 (20.1%)	41.77 (1)	<0.001
No, *n* = 197 (34.4%)	107 (54.3%)	90 (45.7%)		
**Hostility Perception**	**Witnessed violence—*n* (%)**	**Have not witnessed violence—*n* (%)**	**χ^2^ (df)**	***p***
Yes, *n* = 376 (65.6%)	314 (83.5%)	62 (16.5%)	26.44 (1)	<0.001
No, *n* = 197 (34.4%)	127 (64.5%)	70 (35.5%)		

## Data Availability

The data presented in this study are available upon reasonable request to the corresponding author.

## Abbreviations

The following abbreviations are used in this manuscript:

WHO	World Health Organization
ILO	International Labor Organization
PSI	Public Services International
COREN	Regional Nursing Council
UFSC	Federal University of Santa Catarina
ICF	Informed Consent Form
SUS	Brazilian Unified Health System
SPSS	Statistical Package for the Social Sciences
